# Clustering Performances in Elite Basketball Matches According to the Anthropometric Features of the Line-ups Based on Big Data Technology

**DOI:** 10.3389/fpsyg.2022.955292

**Published:** 2022-07-11

**Authors:** Xiao Xu, Mingxin Zhang, Qing Yi

**Affiliations:** ^1^China Basketball College, Beijing Sport University, Beijing, China; ^2^School of Physical Education and Sport Training, Shanghai University of Sport, Shanghai, China

**Keywords:** FIBA Basketball World Cup, match analysis, line-up, anthropometric features, big data technology, clustering performances

## Abstract

The aims of this study were: 1) to conduct a descriptive analysis of the anthropometric features of the line-ups of strong teams (top 16) in the 2019 FIBA Basketball World Cup; 2) to group the line-ups mentioned above into different clusters based on their average height, weight, and body mass index (BMI); and 3) to explore the performance variables that discriminate between various line-up clusters. The play-by-play statistics were collected from 104 team objects in 67 games and 525 line-ups were analyzed using two-step cluster and discriminant analysis. Line-ups were classified into four groups: low average height and weight with middle BMI (LowH–LowW–MiddleBMI); high average height and low average weight with low BMI (HighH–LowW–LowBMI); low average height and high average weight with high BMI (LowH–HighW–HighBMI); high average height and weight with middle BMI (HighH–HighW–MiddleBMI). The results of the discriminant analysis demonstrated that LowH–LowW–MiddleBMI line-ups had the least time played and the lowest offensive rating, but the best offensive rebounds, turnovers, and fastest game pace performance; HighH–LowW–LowBMI line-ups demonstrated the best defensive rating but performed poorly with a low value of assists and a high value of turnovers; the LowH–HighW–HighBMI group achieved the best time played statistics but had the lowest number of free throws made; the HighH–HighW–MiddleBMI group had a higher number of assists and a higher offensive rating and 2-point field goal performance, while also achieving the lowest number of offensive rebounds and ball possessions. These results provide novel insights for coaches and performance analysts to better understand the technical characteristics of different line-ups in elite basketball competitions.

## Introduction

Basketball is one of the most popular sports worldwide, and the academic research on technical performance is well documented at both the team and player levels. The selection of line-ups is a challenging task for coaches during match preparation and match play (Ahmadalinezhad et al., 2019), and choosing an efficient line-up, considering the circumstances of the match, has a direct impact on a team’s match performance. To choose the best line-up, coaches must understand the key factors that affect the line-up’s performance (Gradinaru, 2014; Cui et al., 2019; Zhang et al., 2020; Dong et al., 2021). In a basketball game, coaches can complete line-up adjustments by replacing players during timeouts, dead balls, and other opportunities. Factors affecting line-up efficiency include a player’s position, height and weight, technical characteristics, tactical demands, physical status, etc. (Ahmadalinezhad et al., 2019; Zhang et al., 2020). Therefore, line-up adjustment is an essential game method in the on-the-spot coaching process for basketball coaches. The FIBA Basketball World Cup is one of the most popular basketball competitions in the world, where elite basketball players from various countries compete against each other for the title of world champion. Analyzing this competition is useful for performance analysts and coaches in gaining a deeper understanding of the new trends of technical and tactical behaviors in basketball. Therefore, modeling of game behaviors during this competition has been conducted to observe the relation between behaviors and performance or success, in order to optimize the training and coaching processes.

Players’ anthropometric attributes are an important aspect to consider during performance analysis. Multiple studies have reported that the height, weight, and body mass index (BMI; a value derived from the mass (weight) and height of a person) of players were associated with team performance (Nikolaidis et al., 2015; Teramoto and Cross, 2018; Zhang et al., 2018; Zarić et al., 2020). The playing positions of players are traditionally identified according to their height and weight, meanwhile, the technical and tactical demands of players also including appropriate anthropometric characteristic. Previous studies also showed that there was a significant BMI difference between players of different positions (Alejandro et al., 2015). Back-court players tend to have lower height, weight, and BMI, while post players are taller, heavier, and fatter (Latin et al., 1994; Sallet et al., 2005; Ostojic et al., 2006; Sisodiya and Yadaf, 2010; Alejandro et al., 2015; Nikolaidis et al., 2015). The technical, physical, and physiological characteristics of players differ between the five playing positions (Ostojic et al., 2006; Sampaio et al., 2006). Some research demonstrated that taller players are in short supply and they still have a critical effect on game success (Berri et al., 2005). However, there is a tendency toward what is called a “small-ball line-up” in basketball, which opposes the traditional notion that the height of players is a critical factor in basketball, and instead emphasizes fast game pace, opening court space, and more three-point field goal shooting (Bourbousson et al., 2010; Zhang et al., 2017; Teramoto and Cross, 2018). Due to this, players with a greater height and weight may be losing their importance and attraction from the perspective of coaches.

As the “small ball line-up” trend has drawn lots of attention, there has been more research into how to build an efficient line-up. Some researchers focused on modeling or predicting line-up efficiency according to players’ technical statistics (Ahmadalinezhad et al., 2019; Grassetti et al., 2019; Kalman and Bosch, 2020), while some scholars concentrated on the relationship between line-up players’ positions and the roles they actually played on the court (Sallet et al., 2005; Kalman and Bosch, 2020). Both player efficiency and position setting are affected by the player’s anthropometric features (Drinkwater et al., 2008; Gradinaru, 2014; Zhang et al., 2018). Additionally, the obvious differences between “small ball line-up” and “traditional line-up” are not only the technical and tactical factors (such as 3-point shooting ability, transition offense ability), but also the anthropometrical factors (Szalontai, 2014), which are worthy of further study. However, compared to studies focusing on player and team performance, research on line-up anthropometric attribute analysis is relatively rare. As we all know, at any given moment in a basketball game, there are always five players from each team on the court, making basketball, centrally, a teamwork sport. Players are inevitably influenced by their teammates and the line-up structure (Maymin et al., 2013). Changing the line-up also changes the flow of the game and the eventual outcome.

The FIBA Basketball World Cup provides an excellent opportunity to test the impact of the anthropometric attributes of different line-ups on the match performance of basketball teams. In this case, the current study focuses on the line-up performance of strong teams in the 2019 FIBA Basketball World Cup according to their line-ups’ anthropometric features. We took the line-up height and weight (mean of five players’ height and weight) into account and collected all the line-up combination data to analysis. Additionally, body mass index (BMI) was included as an indicator to illustrate players’ body mass and adipose tissue percentage. With these attributes, we could better describe the anthropometric features of the line-ups and classify line-ups into different groups to explore their statistical differences.

Based on the above details, the aims of our study were: (1) to conduct a descriptive analysis of the anthropometric features of the line-ups of strong teams (top 16) in the 2019 FIBA Basketball World Cup; (2) to group line-ups into different clusters based on their average height, weight, and body mass index (BMI); and (3) to explore the performance variables that discriminate between various line-up clusters.

## Materials and Methods

### Sample and Variables

A descriptive analysis was conducted in this study (Teramoto and Cross, 2010). Player’s height was obtained from the official open-access FIBA World Cup Website^1^. The weight of players was collected from the GameLog program made by the Chinese Basketball Association, the original data of GameLog was come from the Sportradar Company^2^. BMI was calculated based on the following formula: BMI = weight/(height^2^).

The line-up related match statistics were collected using the Advanced Statistics Collection system, a high-level basketball game data analysis system jointly created by the Institute of Computer Science of the Chinese Academy of Sciences and the basketball data analysis team of Beijing Sport University. The ASC system allowed users to generate play-by-play stats based on game videos. The play-by-play statistics of 104 team objects in 67 games played by the top 16 teams were collected. In all games, the teams had completed 1819 line-up uses (the number of line-up appearances), and a total of 1168 line-up types were generated. The line-up statistics were transformed into 40-minute statistics (original stats/min*40) to compare different line-ups in the same dimension (Kubatko et al., 2007). We excluded line-ups that played less than five ball possessions from the sample, considering that those who played only few possessions or played in the 2019 FIBA World Cup for only a short time might generate unreliable transformed statistics (Kubatko et al., 2007). Finally, the sample was limited to 525 line-ups and 6996 ball possession records.

Based on the observational design, this study conformed to the ethical guidelines of the authors’ affiliated institutions through the de-identification of the analyzed data and the disclosing of the data for public scrutiny. However, the data acquisition and application in this study were not ethically approved since FIBA placed no stipulations or restrictions on data reuse for scientific inquiry.

### Reliability and Validity of Data

Based on the available literature (Gradinaru, 2014; Teramoto and Cross, 2018; Cui et al., 2019), the variables shown in Table 1 were selected for our analysis. In order to evaluate the reliability of the data, video recordings of 10 randomly selected games were observed together by four experienced basketball analysts. The results were compared to those collected from the Advanced Statistics Collection system. A high level of agreement (ICC = 0.98) was obtained for most statistics. A slightly different but very acceptable level of agreement (ICC = 0.90) was obtained for the line-up fouls and steals.

**TABLE 1:** Operational definitions of 24 variables selected for the analysis.

Groups	Variable	Abbreviation	Definition
Anthropometric attributes	Line-up height	LH	The average height of line-up players.
	Line-up weight	LW	The average weight of line-up players.
	Body mass index	BMI	The average BMI of line-up players. BMI = weight/(height^2^)
Technical variables	Time played	TP	The number of minutes a line-up played overall.
	2-point field goals scored	2PM	The number of 2-point field goals successfully made by a line-up.
	2-point field goals missed	2PMs	The number of 2-point field goals missed by a line-up.
	3-point field goals scored	3PM	The number of 3-point field goals successfully made by a line-up.
	3-point field goals missed	3PMs	The number of 3-point field goals missed by a line-up.
	Free throws made	FTM	The number of free throws successfully made by a line-up.
	Free throws missed	FTMs	The number of free throws missed by a line-up.
	Offensive rebounds	OREB	The number of rebounds a line-up caught during the offensive part.
	Defensive rebounds	DREB	The number of rebounds a line-up caught during the defensive part.
	Assists	AST	The number of passes that directly lead to a field goal being scored by teammate.
	Turnovers	TOV	The number of losses of offensive ball possession to the defense.
	Steals	STL	The number of interceptions of passes or dribbling in the defense.
	Blocks	BLK	The number of field goal blocks by a defenser.
	Line-up fouls	LF	The number of fouls that line-ups committed.
	Fouls received	FM	The number of fouls drawn from the opposition.
Possession variables	Ball possessions	BP	The number of times that a ball was under control.
	Offensive rating	ORTG	Number of points that a line-up achieved in 100 possessions.
	Defensive rating	DRTG	Number of points that a line-up lost in 100 possessions.

### Statistical Analysis

According to the height, weight, and BMI of the line-ups, we used two-step cluster with log-likelihood as the distance measure and carried out Schwartz’s Bayesian criterion to classify line-ups into four groups (Gómez et al., 2017; Zhang et al., 2017). Then, discriminant analysis was used to determine which statistical variables created the greatest divergence between different line-up clusters. The discriminant analysis model fits these derived rate variables robustly (Cohen et al., 2014). Variables with structure coefficient values >| 0.30| are considered critical factors for the discriminant functions that significantly separate line-up groups (Hoyt et al., 2008). In classifying new data, we used the leave-one-out method of cross-validation to evaluate the usefulness of discriminant functions. The analyzes were performed using the statistical software IBM SPSS for MAC, version 26 (IBM Corp., Armonk, NY, United States). Statistical significance was set at 0.05. The visualization was conducted using Tableau software (Tableau Desktop version 2020.3).

## Results

**FIGURE 1:**
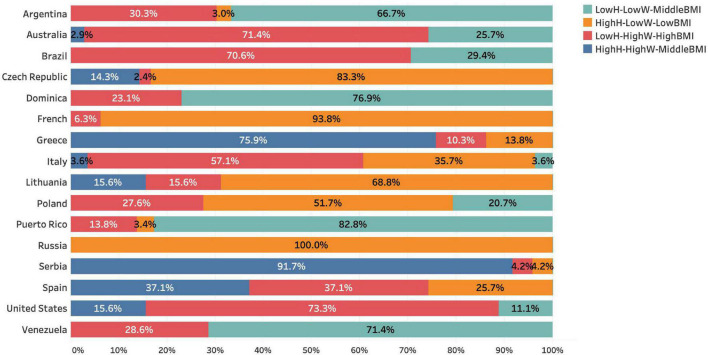
The distribution of line-ups from different national teams within four cluster groups. Note: LowH–LowW–MiddleBMI: low average height and weight with middle BMI; HighH–LowW–LowBMI: high average height and low average weight with low BMI; LowH–HighW–HighBMI: low average height and high average weight with high BMI; HighH–HighW–MiddleBMI: high average height and weight with middle BMI.

The results of the two-step cluster analysis showed that four groups of line-ups were obtained: low average height and weight with middle BMI (LowH–LowW–MiddleBMI); high average height and low average weight with low BMI (HighH–LowW–LowBMI); low average height and high average weight with high BMI (LowH–HighW–HighBMI); high average height and weight with middle BMI (HighH–HighW–MiddleBMI). Firstly, the statistics of the average height, weight, and BMI of four groups and the line-up distribution between the top 16 teams are shown in Table 2. Secondly, a description of the proportion of the 4 cluster groups of the 16 teams in the 2019 FIBA Basketball World Cup is provided to illustrate the constitution of the line-ups of the strong national teams (Figure 1). Lastly, all of the means and standard deviations of 18 variables for 4 clusters of line-ups are presented in Table 3, as well as the structure coefficients for two functions of variables.

**TABLE 2:** Height, weight, and BMI in different line-up groups.

	Cluster1 LowH–LowW– MiddleBMI	Cluster2 HighH–LowW– LowBMI	Cluster3 LowH–HighW– HighBMI	Cluster4 HighH–HighW– MiddleBMI
Total: *n* = 525	*n* = 112	*n* = 156	*n* = 158	*n* = 99
Height (cm)	194.53 ± 1.90	200.41 ± 1.87	198.43 ± 1.92	203.52 ± 1.48
Weight (kg)	93.98 ± 2.03	94.55 ± 2.17	98.39 ± 1.50	101.34 ± 2.16
BMI (kg/m^2^)	24.76 ± 0.57	23.49 ± 0.44	24.96 ± 0.58	24.40 ± 0.42

*Descriptive statistics were presented as mean ± standard deviation.*

**TABLE 3:** Discriminant analysis of different line-up groups.

Clusters/Variables	Cluster1 LowH–LowW– MiddleBMI	Cluster2 HighH–LowW– LowBMI	Cluster3 LowH–HighW– HighBMI	Cluster4 HighH–HighW –MiddleBMI	Function 1 49.9%	Function 2 30.7%
TP 2PM 2PMs 3PM 3PMs FTM FTMs OREB DREB AST TOV STL BLK LF FM BP ORTG DRTG	5.39 ± 4.83 39.67 ± 19.59 21.80 ± 18.73 8.73 ± 10.37 17.26 ± 11.84 20.04 ± 21.11 5.70 ± 8.70 9.60 ± 10.36 25.50 ± 13.82 14.03 ± 11.63 13.08 ± 10.94 8.51 ± 9.60 2.97 ± 5.79 21.25 ± 13.29 22.76 ± 15.42 80.72 ± 17.63 108.77 ± 40.92 109.29 ± 48.63	6.82 ± 9.78 35.73 ± 18.81 17.02 ± 14.95 8.41 ± 8.95 15.45 ± 13.18 16.85 ± 25.56 5.42 ± 9.40 8.23 ± 9.24 25.68 ± 15.33 13.96 ± 11.64 15.85 ± 15.24 8.40 ± 10.74 3.13 ± 6.04 21.06 ± 16.17 21.22 ± 18.00 77.23 ± 21.82 111.59 ± 46.65 94.14 ± 46.48	7.55 ± 9.78 35.98 ± 18.39 16.65 ± 11.65 8.67 ± 10.68 17.08 ± 11.41 13.96 ± 18.42 3.68 ± 6.27 7.20 ± 9.12 27.73 ± 14.86 15.15 ± 11.23 14.73 ± 13.21 7.41 ± 8.39 2.72 ± 5.29 21.00 ± 42.11 18.37 ± 13.41 77.19 ± 26.47 109.27 ± 46.20 106.37 ± 51.25	6.9 ± 8.0 33.63 ± 15.43 15.61 ± 11.17 9.17 ± 8.57 14.55 ± 10.28 17.37 ± 19.31 4.53 ± 6.64 6.23 ± 7.37 25.78 ± 13.58 20.13 ± 12.95 13.11 ± 12.75 7.32 ± 8.59 2.70 ± 4.54 17.68 ± 11.85 21.14 ± 13.35 74.09 ± 13.48 123.34 ± 49.74 110.42 ± 51.59	0.088 –0.045 –0.252 0.089 –0.126 –0.05 –0.126 –0.349 0.039 0.667 –0.193 –0.165 –0.099 –0.240 –0.078 –0.245 0.331 0.34	–0.344 –0.098 0.622 0.037 0.206 0.337 0.206 0.349 0.099 –0.093 –0.308 0.088 –0.004 0.039 0.282 0.308 –0.107 0.385

*Means ± standard deviations and structure coefficients (SC) of line-up performance for four clusters.*

The top 16 teams had 525 line-ups overall (offensive possessions>5) in the 2019 FIBA Basketball World Cup. The average line-up height ranged from 189.2 to 208.4 cm; the average line-up weight ranged from 86 to 107.8 kg; the mean BMI of the players ranged from 22.1 to 26.6 kg/m^2^. The cluster distribution of these 525 line-ups were as follows: 112 of these were in the LowH–LowW–MiddleBMI group; 156 line-ups were included in the HighH–LowW–LowBMI group; 158 line-ups belonged to the LowH–HighW–HighBMI group; the minimum number of line-ups was 99 in the HighH–HighW–MiddleBMI group.

The discriminant analysis revealed 3 functions, but only the first two were significant. The first function (explained 49.9% total variance) and second function (explained 30.7% total variance) accounted for 80.6% of the cumulative variance. Function 1 emphasized two variables that hold structure coefficients >| 0.30| : AST (SC = 0.667) and OREBs (SC = –0.349). Function 2 placed a significant emphasis on 6 variables: 2PMs (SC = 0.622); DRTG (SC = 0.385); TP (SC = –0.344); BP (SC = 0.308); TOV (-0.308); FTM (SC = 0.337).

### Discriminant Variables From Each Cluster Group

The low average height and weight with middle BMI (LowH–LowW–MiddleBMI) group was optimally associated with a number of the explained variables (free throws made, 2-point field goals missed, ball possessions, turnovers and offensive rebounds). This group also had the lowest association with two of the explained variables (offensive rating and time played). The high average height and low average weight with low BMI (HighH–LowW–LowBMI) group had the best association with the discriminant variable defensive rating and the lowest association with two variables (assists and turnovers) from both functions. The low average height and high average weight with high BMI (LowH–HighW–HighBMI) group presented the highest association with the explained variable time played and the lowest association with the free throws made variable. The high average height and weight with middle BMI (HighH–HighW–MiddleBMI) group had the best association with two of the explained variables (assists and offensive rating); meanwhile, this group also presented the lowest association with a number of explained variables (offensive rebounds, ball possessions, defensive rating and 2-point field goals missed).

## Discussion

Compared to research focused on the performance analysis of basketball teams and player research on teammate interactions and coordination, the line-up performance has been studied to a lesser extent (Eccles and Tenenbaum, 2004; Ward and Eccles, 2006; Jackson et al., 2007; Eccles and Johnson, 2008; Poizat et al., 2009). As a middle level between the player and the team, line-up analysis has high academic value and a number of practical applications (Nikolaidis et al., 2015; Ahmadalinezhad et al., 2019; Kalman and Bosch, 2020). The purpose of this study was to examine the effects of anthropometric features on line-up performance in the 2019 FIBA Basketball World Cup. Four categories were obtained according to the average height, weight and BMI of the line-ups. With the results of the discriminant analysis, the performance variables that discriminate the various line-up clusters were identified.

Team line-up changes in multi-player ball games significantly affect the dynamics of game progression and the teams’ performance (Maymin et al., 2013; Oh et al., 2015; Varela-Quintana et al., 2018). As a result, it can be inferred that different line-ups have special functions in various situations that may arise during matches. We focused on the effect of the line-up’s anthropometric features on team performance because the team’s anthropometric attributes (especially player height, weight, and BMI) have drawn lots of attention in basketball performance analysis, along with the popularity of the “small-ball” concept (Teramoto and Cross, 2018; Zarić et al., 2020). Zhang et al. (2018) classified players into different groups according to players’ height, weight, and experience. Teramoto and Cross (2018) used the average height of players weighted by playing time to examine line-up and team height. However, these features probably cannot be used to investigate the anthropometric features of line-ups appropriately. In order to explore the influence of anthropometric factors on line-up performance, the mean of five-man height, weight, and BMI of all the line-up combinations were taken into account. To the best of our knowledge, this study was the first to systematically explore the association between the anthropometric factors of line-ups and line-up performance in international basketball games, and might inspire other researchers to study similar areas in this field, while helping coaches to understand the features of different kinds of line-ups.

The LowH–LowW–MiddleBMI group included line-ups that had a lower average height and weight, but a mid-level BMI. In this case, we can assume that LowH–LowW–MiddleBMI line-ups are the line-ups with the shortest heights. Of the 525 line-ups, 112 short-height line-ups (those who had played more than five offensive possessions) participated in the 2019 FIBA Basketball World Cup; interestingly, only 42 short-height line-ups were used by the top eight teams (line-up proportion is presented in Table 1 and Figure 1). Additionally, the average time played for this type of line-up was only 5.39 ± 4.83 min, the lowest amount of time played out of the four groups. As such, it does not appear as though the top eight teams consider using low height and weight line-ups very often. Players’ heights and weights play an important role in team performance, due to the advantages brought by their physical condition and the control of higher space brought by their height, which cannot be improved by training (Zhang et al., 2018; Zarić et al., 2020). The LowH–LowW–MiddleBMI line-ups had the best association with the following discriminant variables: ball possessions, 2-point field goals missed, offensive rebounds, and number of free throws made. The evidence presented by Garcia-Rubio et al. (2014) and Ferioli et al. (2018) demonstrated that the agility, endurance, and drilling ability of forwards and guards were better than those of centers. This supports our belief that LowH–LowW–MiddleBMI line-ups achieve more ball possessions and enable a faster game pace (Oliver, 2004). This kind of line-up also had the highest number of 2-point field goals, combined with the fastest game pace, excellent dribbling, and layup skill in low height and weight players (Drinkwater et al., 2008), so we can assume that the LowH–LowW–MiddleBMI line-ups had a better transition performance. Despite their good scoring efficiency during transitions compared to other scoring methods (Matulaitis and Bietkis, 2021), LowH–LowW–MiddleBMI line-ups achieved the lowest offensive rating and the highest 2-point field goal missed. In this case, this group was the only one that had a negative rating (as a result of their offensive rating minus their defensive rating). There are three reasons which might explain this. Firstly, the fastest players with are more likely to commit more turnovers (Gryko et al., 2020); secondly, when this type line-ups are forced into half-court offense, the speed advantage is weakened and the opponent’s defense with higher and weightier players is difficult to break; thirdly, in terms of height and weight factors, post players with higher heights and weights were more threatening near the basket, which could make for better offensive efficiency for the opponent. However, this group achieved the lowest 2-point field goal percentage out of all four groups, and previous research showed that 2-point ability was a key factor in winning games (Lorenzo et al., 2010; Gryko et al., 2018). Though some researchers stated that team height was not a significant predictor of winning games in the NBA (Teramoto and Cross, 2018), there still are papers that show that player size played an essential role in team performance (Drinkwater et al., 2008). Due to its low offensive rating and the lowest amount of time played, a low height and weight line-up may not be a kind of good regular line-up, but be a functional choice for international basketball competitions. On the one hand, LowH–LowW–MiddleBMI line-ups can make more offensive transitions and enable a faster game pace. On the other hand, these kinds of line-ups have a better dribbling and free throw ability, which means that LowH–LowW–MiddleBMI line-ups perform well when a coach needs to set a press defense or react to a foul strategy in a crucial moment by introducing more players with an excellent free throw ability to the court.

The HighH–LowW–LowBMI (156 of 525) cluster contained line-ups with a high height, low weight, the lowest BMI. With this low weight and BMI, we can regard this group as a lightweight and forward line-up, which could be considered as a new-type of line-up in the development of basketball. Since the enhancement of training and player selection (Skinner and Guy, 2015), it has been common to see talented players mastering the skills of different positions and doing more work than before, such as LeBron James, Giannis Antetokounmpo and Kevin Durant in the National Basketball Association (NBA). In consideration of their excellent ability, we can hardly define these players by one single playing position. Therefore, some researchers believe that basketball should be regarded more as a “position-less” sport (Kalman and Bosch, 2020), or that players should be classified into various categories based on their ability and role (Alagappan, 2012). In particular, forward players are more versatile than players in other positions according to their talent and anthropometric features (Rangel et al., 2019). The high height and low weight with low BMI cluster showed the best association with two of the discriminant variables (defensive rating and turnovers) for both functions. This cluster achieved the best defensive rating (94.142 points lost in 100 ball possessions) and the steal and block statistics might give us an explanation for this. With a lower weight and BMI, players in this line-up can move more quickly and make more switches or press defense (Nikolaidis et al., 2015); with a higher height, players can control a higher space (Teramoto and Cross, 2018). With the best defense rating and a good offensive rating (second place out of the four clusters), this cluster had the best net rating. Compared to other groups, the excellent rating performance of HighH–LowW–LowBMI line-ups highlight that movement ability and agility might play a more important role in modern basketball, which emphasizes transition efficiency and dynamic offense and defense (Yano et al., 2020). Lastly, coaches who want to use a HighH–LowW–LowBMI line-up should consider controlling their line-up’s turnovers to enable a better performance.

The LowH–HighW–HighBMI (159 of 525) group included line-ups with a low height, a high weight, and a high-level BMI. This group had the best association with the discriminant variable time played and the lowest association with the free throws made variable in both functions. A notable feature of this group was that its BMI was highest out of all of the groups, while its average height was a low level (second place) out of all of the groups. Obviously, because of their anthropometric features, LowH–HighW–HighBMI line-ups can hardly employ a fast game pace (the number of ball possessions they achieved put them in third place). Moreover, the fact that this group achieved the lowest number of free throws and fouls received meant that this cluster lacked an impact force toward their opponents. Previous studies demonstrated that body mass status may influence players’ running and jumping performance (Ostojic et al., 2006; Nikolaidis et al., 2015); therefore, we assume that the reason for the slow pace and lack of an impact may not be due to the average height of the line-up, but to the line-up’s weight and BMI.

The HighH–HighW–MiddleBMI (99 of 525) group comprised line-ups with high heights and weights, and mid-level BMIs. Some line-ups of this type had 3 or even 4 power forwards and centers on the court. The HighH–HighW–MiddleBMI line-ups had the best association with three of the discriminant variables (assists, offensive rating and defensive rating) from both functions, as well as the lowest association with several of the explained variables (ball possessions, offensive rebounds and 2-point field goals missed). This was surprising, as we had expected that, with the “small-ball” style being popular worldwide, the number of big-man line-ups might be reduced. However, the highest height and weight line-ups were more often used by the top eight teams (71 of 99) with the best mean offensive rating (123.34 points in 100 possessions). HighH–HighW–MiddleBMI line-ups had the best assist and the second-best turnover performance, which are considered key factors in winning a game according to previous studies (Mori et al., 2002; Gómez et al., 2008; Lorenzo et al., 2010). A possible explanation could be associated with the ball possessions and game pace of this line-up, as high height and weight players carried out more half-court offense rather than transitions (García et al., 2020). The threat of post players was so critical that coaches would arrange for more help defense, then inside-out passes would happen and result in assists (Drinkwater et al., 2008; Koon Teck et al., 2012). Additionally, fewer transitions and higher space control ability mean fewer turnovers (Teramoto and Cross, 2010). Besides their assist and turnover performance, these line-ups’ remarkable field goal percentage also contributed to them achieving the best offensive rating. The HighH–HighW–MiddleBMI group achieved first place in terms of their 3-point field goal percentage and second place for their 2-point field goal percentage, which means that these line-ups possessed excellent shooting skills.

Our impression is that bigger players do not seem to be as good at shooting, especially from middle and long distances (Teramoto and Cross, 2010; Nakano et al., 2020). Two possible explanations for this matter may be as follows: firstly, as centers and power forwards have more offensively threaten than back-court players in post, it is easier for them to score 2-point field goals; secondly, with the development of basketball tactics, a trend has arisen in which big players’ shooting skills have become increasingly important, and it is no surprise to see centers accurately shooting from outside of the 3-point line (Zhang et al., 2017; Chang, 2018). Based on our results, 13 centers shot at least one 3-point field goal per game, with an excellent shooting percentage in the top eight teams. This evidence helps us to understand why the top eight teams used more HighH–HighW–MiddleBMI line-ups.

What beyond our expectation is the rebound performance in this study. Rebound is a key indicator of a winning game according to other research (Gómez et al., 2008). However, the defensive rebounds statistic did not differ significantly between the four categories (the structure coefficient absolute value of defensive rebounds is smaller than 0.3). In the aspect of offensive rebounds, the lowest-height cluster (LowH-LowW-MiddleBMI) showed the best performance in terms of their OREBs. We assume that the reason for this might be complicated. One possible explanation would be the fact that taller players have fewer rebounds than before because of further rebounds caused by the increase of long-distance shots, so guards and forwards may catch rebounds (especially offensive rebounds) more easily (Okubo and Hubbard, 2015). Other suitable explanations could be explored in future studies.

As mentioned above, the four clusters exerted various features that might help us to gain a better understanding of some of the hotspots of basketball analysis, the first of which is the “small-ball” style. Since the Golden State Warriors won three NBA championships in the 2015-2018 seasons, the “small-ball” style has attracted worldwide attention (Teramoto and Cross, 2018; Ahmadalinezhad et al., 2019; Zhang et al., 2020). This style favors speed, open space, transition attacks, and the use of the three-point shot in the offense and a flexible switch defense (Ahmadalinezhad et al., 2019). In our study, the LowH–LowW–MiddleBMI and HighH–LowW–LowBMI groups were considered in this way as they partially performed as small-ball line-ups by emphasizing movement ability and shooting skill. The LowH–LowW–MiddleBMI line-ups had the fastest game pace and the most 3-point field goal attempts. The HighH–LowW–LowBMI line-ups had the best defensive rating, the second-best ball possessions, and the second-best field goal percentage. Combined with the evidence of previous studies (Berri et al., 2005; Sallet et al., 2005), we assumed that low-weight and fully skilled players, especially forward players, might key factors in small-ball line-ups. Secondly, the “small-ball” trend questions the perceived importance of height in basketball (Schwartz, 2015). However, according to our analysis and other studies (Berri et al., 2005; Bourbousson et al., 2010; Zhang et al., 2017; Kalman and Bosch, 2020), team height might be underestimated. Line-ups with the greatest height (HighH–HighW–MiddleBMI line-ups) achieved the best offensive performance in the 2019 FIBA Basketball World Cup. This illustrates that big players with superior skills can compose a threatening line-up. In this case, a high-height line-up is still a good choice for international team coaches.

## Limitations and Future Directions

This study provided a new way in which to understand the influence of line-ups’ anthropometric features on their technical performance. However, the limitations of the current study should be noted. Firstly, as we were restricted by time and workload, we only analyzed the 2019 FIBA Basketball World Cup. In order to obtain better results, a larger line-up sample size is necessary. Secondly, contextual factors (such as opponent line-up features, score differences, and so forth) should be considered in further research to explore the line-up performance in different game situations. Thirdly, due to the difficulty of data collection, only mean of five players’ heights, weights, and BMIs were analyzed in our study as lineup characteristics, all three of which are basic factors of anthropometric features. Indicators such as arm length and shoulder width have also been shown to be important factors affecting the play of basketball players, while the impact of anthropometric features varies among players at different positions, suggesting that there is still potential for continued in-depth research on line-ups’ anthropometric feature. Hence, in the future, more anthropometric indicators of line-ups and the distribution of players’ individual anthropometric attributes within their line-ups could also be a promising research direction. This study can help coaches better understand the feature of line-ups, but in coaching applications, coaches should realize that selecting players and setting lineups should also take into account both the game context as well as the players’ technical and tactical abilities and the chemistry of the players in the lineups. Future research could provide an in-depth analysis of the interaction of line-ups’ anthropometric characteristics with context factors, players’ technical and tactical features to conduct a better application value.

## Conclusion

In summary, four different line-up groups were obtained by cluster analysis. LowH–LowW–MiddleBMI line-ups had the least time played and the lowest offensive rating but the best offensive rebounds, the lowest turnover value, and the fastest game pace performance; HighH-LowW-LowBMI line-ups demonstrated a higher defensive rating, but did not perform as well in terms of assists and turnovers; the LowH–HighW–HighBMI group achieved the best time played statistics, but the lowest number of free throws made; the HighH–HighW–MiddleBMI group had a higher number of assists and a higher offensive rating and 2-point field goal performance, while also achieving the lowest number of offensive rebounds and ball possessions. These results provide novel insights for coaches and performance analysts to better understand the technical characteristics of different line-ups in elite basketball competitions. Different line-up groups serve various functions on the basis of their anthropometric attributes. This appears to underline the fact that line-up height and weight still play an essential role in modern basketball competitions against the backdrop of the small-ball concept. Our findings may be helpful for coaches when recruiting players, planning training projects, and setting game strategies according to the type and function of their line-ups.

## Data Availability Statement

The original contributions presented in this study are included in the article/Supplementary Material, further inquiries can be directed to the corresponding author.

## Author Contributions

XX, MZ, and QY: conceptualization and supervision. QY: methodology, software, data curation, and visualization. XX and MZ: validation and writing—review and editing. QY: formal analysis and writing—original draft preparation. MZ: funding acquisition. All authors have read and agreed to the published version of the manuscript.

## Conflict of Interest

The authors declare that the research was conducted in the absence of any commercial or financial relationships that could be construed as a potential conflict of interest.

## Publisher’s Note

All claims expressed in this article are solely those of the authors and do not necessarily represent those of their affiliated organizations, or those of the publisher, the editors and the reviewers. Any product that may be evaluated in this article, or claim that may be made by its manufacturer, is not guaranteed or endorsed by the publisher.

## Footnotes

1
http://www.fiba.basketball

2
https://sportradar.com

## Supplementary Material

Supplementary Table 1 The anthropometric, technical and tactical data of all the 525 line-ups.

## References

[B1] Ahmadalinezhad M. Makrehchi M. Seward N. (2019). “Basketball lineup performance prediction using network analysis,” in *Proceedings of the 2019 IEEE/ACM International Conference on Advances in Socialnetworks Analysis and Mining*, (Vancouver). 519–524. 10.1145/3341161.3342932

[B2] Alagappan M. (2012). “Redefining the positions in basketball,” in *Proceedings of MIT Sloan Sports Analytics Conference*, (Boston, MA).

[B3] Alejandro V. Santiago S. Gerardo V. J. Carlos M. J. Vicente G.-T. (2015). Anthropometric characteristics of Spanish professional basketball players. *J. Hum. Kinet.* 46:99. 10.1515/hukin-2015-0038 26240653 PMC4519226

[B4] Berri D. J. Brook S. L. Frick B. Fenn A. J. Vicente-Mayoral R. (2005). The short supply of tall people: competitive imbalance and the National Basketball Association. *J. Econ. Issues* 39 1029–1041. 10.1080/00213624.2005.11506865

[B5] Bourbousson J. Poizat G. Saury J. Seve C. (2010). Team coordination in basketball: description of the cognitive connections among teammates. *J. Appl. Sport Psychol.* 22 150–166. 10.1080/10413201003664657

[B6] Chang S.-C. (2018). Capability and opportunity in hot shooting performance: evidence from top-scoring NBA leaders. *PLoS One* 13:e0179154. 10.1371/journal.pone.0179154 29432458 PMC5809017

[B7] Cohen P. West S. G. Aiken L. S. (2014). *Applied Multiple Regression/Correlation Analysis for the Behavioral Sciences.* London: Psychology press. 10.4324/9781410606266

[B8] Cui Y. Liu F. Bao D. Liu H. Zhang S. Gómez M. -Á (2019). Key anthropometric and physical determinants for different playing positions during National Basketball Association Draft Combine Test. *Front. Psychol.* 10:235. 10.3389/fpsyg.2019.02359 31708831 PMC6820507

[B9] Dong R. Lian B. Zhang S. Zhang M. Huang S. Z. O’DONOGHUE P. (2021). Addressing opposition quality in basketball performance evaluation. *Int. J. Perf. Anal. Sport* 21 263–276. 10.1080/24748668.2021.1877938

[B10] Drinkwater E. J. Pyne D. B. Mckenna M. J. (2008). Design and interpretation of anthropometric and fitness testing of basketball players. *Sports med.* 38 565–578. 10.2165/00007256-200838070-00004 18557659

[B11] Eccles D. W. Johnson M. B. (2008). *Letting the Social and Cognitive Merge: New Concepts for an Understanding of Group Functioning in Sport. Advances in Applied Sport Psychology.* Abingdon-on-Thames: Routledge.

[B12] Eccles D. W. Tenenbaum G. (2004). Why an expert team is more than a team of experts: a social-cognitive conceptualization of team coordination and communication in sport. *J. Sport Exer. Psychol.* 26 542–560. 10.1123/jsep.26.4.542

[B13] Ferioli D. Rampinini E. Bosio A. La Torre A. Azzolini M. Coutts A. J. (2018). The physical profile of adult male basketball players: differences between competitive levels and playing positions. *J. Sports Sci.* 36 2567–2574. 10.1080/02640414.2018.1469241 29697296

[B14] García F. Vázquez-Guerrero J. Castellano J. Casals M. Schelling X. (2020). Differences in physical demands between game quarters and playing positions on professional basketball players during official competition. *J. Sports Sci. Med.* 19:256. 32390718 PMC7196749

[B15] Garcia-Rubio J. Ibanez S. J. Gomez M. A. Sampaio J. (2014). Basketball Game-related statistics discriminating ACB league teams according to game location, game outcome and final score differences. *Int. J. Perf. Anal. Sport* 14 443–452. 10.1080/24748668.2014.11868733

[B16] Gómez M. A. Lorenzo A. Barakat R. Ortega E. José M. P. (2008). Differences in game-related statistics of basketball performance by game location for men’s winning and losing teams. *Percept. Mot. Skills* 106, 43–50. 10.2466/pms.106.1.43-50 18459354

[B17] Gómez M. -Á Silva R. Lorenzo A. Kreivyte R. Sampaio J. (2017). Exploring the effects of substituting basketball players in high-level teams. *J. Sports Sci.* 35 247–254. 10.1080/02640414.2016.1161217 26986448

[B18] Gradinaru S. (2014). The most important key-performance indicators at BC Timisoara management. Timisoara *Phys. Educ. Rehabil. J.* 6:13. 10.2478/tperj-2014-0022

[B19] Grassetti L. Bellio R. Fonseca G. Vidoni P. (2019). Estimation of lineup efficiency effects in basketball using play-by-play data. *Book Short Papers* 2019 363–370.

[B20] Gryko K. Mikołajec K. Marszałek J. Adamczyk J. G. Molik B. Waśkiewicz Z. (2020). How did basketball teams win EuroBasket 2015? A non-standard analysis of performance based on passes, dribbling and turnovers. *Int. J. Perf. Anal. Sport* 20 339–356. 10.1080/24748668.2020.1749013

[B21] Gryko K. Mikołajec K. Maszczyk A. Cao R. Adamczyk J. G. (2018). Structural analysis of shooting performance in elite basketball players during FIBA EuroBasket 2015. *Int. J. Perf. Anal. Sport* 18 380–392. 10.1080/24748668.2018.1479923

[B22] Hoyt W. T. Imel Z. E. Chan F. (2008). Multiple regression and correlation techniques: recent controversies and best practices. *Rehab. Psychol.* 53:321. 10.1037/a0013021

[B23] Jackson B. Beauchamp M. R. Knapp P. (2007). Relational efficacy beliefs in athlete dyads: an investigation using actor–partner interdependence models. *J. Sport Exer. Psychol.* 29 170–189. 10.1123/jsep.29.2.170 17568065

[B24] Kalman S. Bosch J. (2020). “NBA lineup analysis on clustered player tendencies: A new approach to the positions of basketball & modeling lineup efficiency of soft lineup aggregates. 42 Analytics (2020),” in *Proceedings of the 14th MIT Sloan Sports Analytics Conference*, (Boston, MA). 1–17.

[B25] Koon Teck K. Wang C. Mallett C. (2012). Discriminating factors between successful and unsuccessful elite youth Olympic female basketball teams. *Int. J. Perform. Anal. Sport* 12 119–131. 10.1080/24748668.2012.11868588

[B26] Kubatko J. Oliver D. Pelton K. Rosenbaum D. T. (2007). A starting point for analyzing basketball statistics. *J. Quant. Anal. Sports* 3 10.2202/1559-0410.1070

[B27] Latin R. W. Berg K. Baechle T. (1994). Physical and performance characteristics of NCAA division Imale basketball players. *J. Strength Cond. Res.* 8 214–218. 10.1519/00124278-199411000-00002

[B28] Lorenzo A. Gómez M. Á Ortega E. Ibáñez S. J. Sampaio J. (2010). Game related statistics which discriminate between winning and losing under-16 male basketball games. *J. Sports Sci. Med.* 9:664. 24149794 PMC3761811

[B29] Matulaitis K. Bietkis T. (2021). Prediction of offensive possession ends in elite basketball teams. *Int. J. Environ. Res. Public Health* 18:1083. 10.3390/ijerph18031083 33530475 PMC7908613

[B30] Maymin A. Maymin P. Shen E. (2013). NBA chemistry: positive and negative synergies in basketball. *Int. J. Comput. Sci. Sport* 12 4–23.

[B31] Mori S. Ohtani Y. Imanaka K. (2002). Reaction times and anticipatory skills of karate athletes. *Hum. Mov. Sci.* 21 213–230. 10.1016/S0167-9457(02)00103-3 12167300

[B32] Nakano N. Fukashiro S. Yoshioka S. (2020). The effect of increased shooting distance on energy flow in basketball jump shot. *Sports Biomech.* 19 366–381. 10.1080/14763141.2018.1480728 30001184

[B33] Nikolaidis P. T. Asadi A. Santos E. J. Calleja-González J. Padulo J. Chtourou H. (2015). Relationship of body mass status with running and jumping performances in young basketball players. *Muscles Ligaments Tendons J.* 5:187. 10.11138/mltj/2015.5.3.187 26605193 PMC4617219

[B34] Oh M.-H. Keshri S. Iyengar G. (2015). “Graphical model for basketball match simulation,” in *Proceddings of the2015 MIT Sloan Sports Analytics Conference*, (Boston, MA).

[B35] Okubo H. Hubbard M. (2015). Rebounds of basketball field shots. *Sports Eng.* 18 43–54. 10.1007/s12283-014-0165-z

[B36] Oliver D. (2004). *Basketball on Paper: Rules and Tools for Performance Analysis.* Sterling, VA: Potomac Books, Inc.

[B37] Ostojic S. M. Mazic S. Dikic N. (2006). Profiling in basketball: physical and physiological characteristics of elite players. *J. Strength Cond. Res.* 20:740. 10.1519/00124278-200611000-00003 17149984

[B38] Poizat G. Bourbousson J. Saury J. Sève C. (2009). Analysis of contextual information sharing during table tennis matches: an empirical study of coordination in sports. *Int. J. Sport Exer. Psychol.* 7 465–487. 10.1080/1612197X.2009.9671920

[B39] Rangel W. Ugrinowitsch C. Lamas L. (2019). Basketball players’ versatility: Assessing the diversity of tactical roles. *Int. J. Sports Sci. Coach.* 14 552–561. 10.1177/1747954119859683

[B40] Sallet P. Perrier D. Ferret J. Vitelli V. Baverel G. (2005). Physiological differences in professional basketball players as a function of playing position and level of play. *J. Sports Med. Phys. Fitness* 45:291. 16230979

[B41] Sampaio J. Janeira M. Ibáñez S. Lorenzo A. (2006). Discriminant analysis of game-related statistics between basketball guards, forwards and centres in three professional leagues. *Eur. J. Sport Sci.* 6 173–178. 10.1080/17461390600676200

[B42] Schwartz M. (2015). Warriors Posting Big Numbers with Small Ball Lineup. Available online at: http://www.espn.com/blog/statsinfo/post/_/id/111870/warriors-posting-big-numbers-with-small-ball-lineup (accessed November 24, 2015).

[B43] Sisodiya A. Yadaf M. (2010). Relationship of anthropometric variables to basketball playing ability. *J. Adv. Dev. Res.* 1 191–194.

[B44] Skinner B. Guy S. J. (2015). A method for using player tracking data in basketball to learn player skills and predict team performance. *PLoS One* 10:e0136393. 10.1371/journal.pone.0136393 26351846 PMC4564231

[B45] Szalontai J. D. (2014). *Small Ball in the Big Leagues: A History of Stealing, Bunting, Walking and Otherwise Scratching for Runs.* McFarland CA: McFarland.

[B46] Teramoto M. Cross C. L. (2010). Relative importance of performance factors in winning NBA games in regular season versus playoffs. *J. Quant. Anal. Sports* 6 1–19. 10.2202/1559-0410.1260

[B47] Teramoto M. Cross C. L. (2018). Importance of team height to winning games in the National Basketball Association. *Int. J. Sports Sci. Coach.* 13 559–568. 10.1177/1747954117730953

[B48] Varela-Quintana C. Del Corral J. Prieto-Rodriguez J. (2018). Home-team response to the three-point victory rule: new evidence using double and triple differences. *Int. J. Sport Finance* 13 353–372.

[B49] Ward P. Eccles D. W. (2006). A commentary on “team cognition and expert teams: emerging insights into performance for exceptional teams”. *Int. J. Sport Exerc. Psychol.* 4 463–483. 10.1080/1612197X.2006.9671808

[B50] Yano S. Matsuura K. Tanioka H. Karungaru S. Kohda N. Gotoda N. (2020). “Tactics-trend analysis for increasing the possibility of shooting in a basketball match,” in *In Proceedings of 2020 14th International Conference on Ubiquitous Information Management and Communication (IMCOM)*, (Piscataway, NJ). 1–4. 10.1109/IMCOM48794.2020.9001784

[B51] Zarić I. Kukić F. Jovićević N. Zarić M. Marković M. Toskić L. (2020). Body height of elite basketball players: do taller basketball teams rank better at the fiba world cup? *Int. J. Environ. Res. Public Health* 17:3141. 10.3390/ijerph17093141 32365985 PMC7246476

[B52] Zhang S. Gomez M. Á Yi Q. Dong R. Leicht A. Lorenzo A. (2020). Modelling the relationship between match outcome and match performances during the 2019 FIBA Basketball World Cup: a quantile regression analysis. . *Int. J. Environ. Res. Public Health* 17:5722. 10.3390/ijerph17165722 32784740 PMC7460061

[B53] Zhang S. Lorenzo A. Gómez M.-A. Liu H. Gonçalves B. Sampaio J. (2017). Players’ technical and physical performance profiles and game-to-game variation in NBA. *Int. J. Perform. Anal. Sport* 17 466–483. 10.1080/24748668.2017.1352432

[B54] Zhang S. Lorenzo A. Gómez M.-A. Mateus N. Gonçalves B. Sampaio J. (2018). Clustering performances in the NBA according to players’ anthropometric attributes and playing experience. *J. Sports Sci.* 36 2511–2520. 10.1080/02640414.2018.1466493 29676222

